# Incorporating N Atoms into SnO_2_ Nanostructure as an Approach to Enhance Gas Sensing Property for Acetone

**DOI:** 10.3390/nano9030445

**Published:** 2019-03-15

**Authors:** Xiangfeng Guan, Yongjing Wang, Peihui Luo, Yunlong Yu, Dagui Chen, Xiaoyan Li

**Affiliations:** 1 Organic Optoelectronics Engineering Research Center of Fujian’s Universities, Fujian Jiangxia University, Fuzhou 350108, China; phluo@fjjxu.edu.cn (P.L.); ylyu@fjjxu.edu.cn (Y.Y.); dgchen@fjjxu.edu.cn (D.C.); xyli@fjjxu.edu.cn (X.L.); 2 College of Environment and Resources, Fuzhou University, Fuzhou 350108, China; yjwang03@fzu.edu.cn

**Keywords:** acetone gas sensor, nitrogen incorporating, mesoporous structure, tin oxide

## Abstract

The development of high-performance acetone gas sensor is of great significance for environmental protection and personal safety. SnO_2_ has been intensively applied in chemical sensing areas, because of its low cost, high mobility of electrons, and good chemical stability. Herein, we incorporated nitrogen atoms into the SnO_2_ nanostructure by simple solvothermal and subsequent calcination to improve gas sensing property for acetone. The crystallization, morphology, element composition, and microstructure of as-prepared products were characterized by X-ray diffraction (XRD), transmission electron microscopy (TEM), scanning electron microscopy (SEM), X-ray photoelectron spectroscopy (XPS), Electron paramagnetic resonance (EPR), Raman spectroscopy, UV–visible diffuse reflectance spectroscopy (UV–vis DRS), and the Brunauer–Emmett–Teller (BET) method. It has been found that N-incorporating resulted in decreased crystallite size, reduced band-gap width, increased surface oxygen vacancies, enlarged surface area, and narrowed pore size distribution. When evaluated as gas sensor, nitrogen-incorporated SnO_2_ nanostructure exhibited excellent sensitivity for acetone gas at the optimal operating temperature of 300 °C with high sensor response (R_air_/R_gas_ − 1 = 357) and low limit of detection (7 ppb). The nitrogen-incorporated SnO_2_ gas sensor shows a good selectivity to acetone in the interfering gases of benzene, toluene, ethylbenzene, hydrogen, and methane. Furthermore, the possible gas-sensing mechanism of N-incorporated SnO_2_ toward acetone has been carefully discussed.

## 1. Introduction

Gas sensor has many important applications such as environment monitoring, industrial and personal safety, and medical diagnostics, etc. In recent years, the indoor pollutions of volatile organic compounds (VOCs) have been much concerned as the inhalation of the slowly released VOCs could induce chronic toxic effects to human health. Acetone is one of the typical VOCs, which has been frequently applied in scientific labs and industries. High concentration of acetone (>173 ppm) can anaesthetize the central nervous system and cause damage to important organs of the human body. Consequently, some major environmental safety agencies including National Institute of Occupational Safety and Health (NIOSH) and European Agency for Safety and Health at Work (EU-OSHA) have established guidelines to limit the exposure of human to acetone in indoor and workplace air [1]. For example, the cumulative exposure limits of acetone gases measured in an average user-adjusted period of 4–16 h is 250 ppm and 1000 ppm prescribed by NIOSH and EU-OSHA, respectively. Therefore, high sensibility with a ppm-level detection limit and good selectivity towards acetone are required for acetone gas sensor applied in indoor and workplace. Among the versatile candidate materials for gas sensor towards acetone, metal oxides such as SnO_2_ [2], WO_3_ [3], ZnO [4], and In_2_O_3_ [5] are most commonly used due to their good sensitivity and low power consumption. In particular, SnO_2_ shows excellent gas sense performance and has been intensively investigated, because of its low cost, high electron conductivity, and good thermal and chemical stability [6,7]. It has been widely known that the sensitivity is an important parameter for the gas sensor, besides other parameters such as selectivity and response-recovery time [8]. The higher sensitivity means better capability to detect the target gas. However, the sensitivity towards acetone of the above-mentioned metal oxides materials is still to be improved for practical applications [9].

One feasible way for increasing the sensitivity is to choose the materials with large surface area to provide more active sites [10]. In this regard, many ingenious synthetic strategies have been used to prepare various types of nanostructured SnO_2_ such as porous hierarchical flower-like nanomaterials [11], hollow microcubes [12], porous microtubules [13], nanorods [14], nanowires [15], hierarchical micro-nanostructures [16]. Theoretically speaking, the improvement of the sensitivity by utilizing large surface area materials is limited as the properties of gas sensors are dependent primarily on the electronic and structural properties of the materials [17]. From this perspective, as a common method for modulating the electronic and structural properties of a semiconductor, the extrinsic ion incorporation is considered as an important competitive method. For instances, Singh et al. synthesized Er-doped SnO_2_ [18] and Gd-doped nanostructures [19] with enhanced sensor response resulted from large surface area and enormous oxygen vacancies [20]. Li et al. pointed that oxygen vacancies were beneficial to the adsorption of surface oxygen species, which was attributed to the enhanced gas sensitivity of Pr-doped SnO_2_ [21]. Gao et al. studied the acetone sensing properties of La-doped SnO_2_ nanoarrays [22]. Patil et al. reported the acetone gas sensing properties of Co-doped SnO_2_ thin films [23].

Despite the tremendous researches on the influence of cationic incorporation on SnO_2_ gas-sensing properties, researches on anion incorporation are rather limited. Guo et al. reported that the sensitivity of SnO_2_ gas sensor towards H_2_ was significantly improved by Fluorine (F) doping because of the increased conductivity [24]. Basu et al. studied the enhanced gas sensing behaviour of F-doped SnO_2_ towards alcohol [25]. When compared with F element, nitrogen (N) is another ideal dopant for SnO_2_ because of the similar ionic radii of nitrogen and oxygen [26]. It is reported that the intrinsic electrical resistance and signal noise level could be well reduced by nitrogen (N)-incorporation of metal oxides [27]. Sun et al. showed that the N-doping would result in a reduction in band gap and it was energetically favourable for substituting O with N [28]. In bulk N-incorporated SnO_2_, significantly enhanced conductivities were observed [29]. However, there is no gas sensor implication for N incorporated SnO_2_ nanostructure and it is still unclear whether N incorporation can effectively enhance the gas-sensing property of SnO_2_.

Here, we report our first step toward this direction and show that the incorporation of nitrogen into the SnO_2_ nanostructure enhances its gas sensing properties towards acetone. The relevant acetone gas-sensing mechanism of N-incorporated SnO_2_ gas sensor was discussed.

## 2. Materials and Methods

### 2.1. Sample Preparation

**Figure 1:**
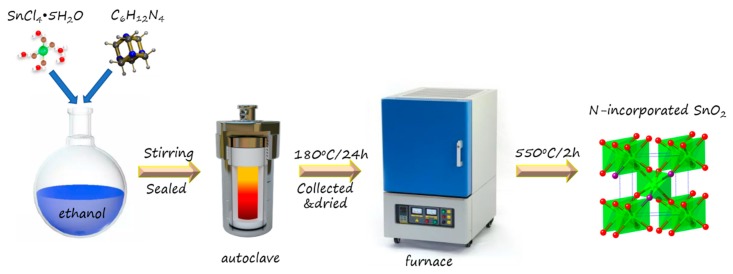
Scheme of the synthesis process of N-incorporated SnO_2_ nanostructure.

In the experiment, the samples were prepared by simple solvothermal and subsequent calcination. For the synthesis of N-incorporated SnO_2_ sample, 1.0518 g SnCl_4_·5H_2_O (Sinopharm Chemical Reagent Co., Ltd, Shanghai, China) was dissolved in 32 mL ethanol (Sinopharm Chemical Reagent Co., Ltd, Shanghai, China) and then 1.2608 g hexamethylenetetramine (C_6_H_12_N_4_, Sinopharm Chemical Reagent Co., Ltd, Shanghai, China) was added to the transparent solution with vigorous stirring, which formed a white suspension. The suspension was put into a 45 mL Teflon-lined stainless-steel autoclave, which was heated at 180 °C for 24 h and cooled naturally. The resultant product was washed by water and ethanol several times, and then dried in air at 80 °C for 12 h. Finally, the dried product was calcined at 550 °C for 2 h to obtain N-incorporated SnO_2_ nanostructure. For the synthesis of pure SnO_2_ sample, no C_6_H_12_N_4_ was used, while the other experiment conditions were the same. The key synthesis process of N-incorporated SnO_2_ nanostructure is shown in Figure 1.

### 2.2. Sample Characterization

X-ray diffraction (XRD, MiniFlex II, Rigaku, Tokyo, Japan) and transmission electron microscopy (TEM, JEM-2010, JEOL, Tokyo, Japan) were used to characterize the phase structures and microstructures of the samples, respectively. Scanning electron microscopy (SEM, JSM-6700-F, JEOL, Tokyo, Japan) and energy dispersive spectrometer (EDS, Oxford INCA Energy 250, Oxford Instruments, Abingdon, UK) were employed to study the elements distribution. X-ray photoelectron spectroscopy (XPS, ESCA-LAB250XI, Waltham, MA, USA) was used to characterize chemical compositions and valence states of the samples with a monochromatic Al Ka X-ray source. Electron paramagnetic resonance (EPR, Bruker-BioSpin E500, Rheinstetten, Germany) was performed to study the defects of the samples. Raman spectroscopy (Horiba, Labram HR800 Evolution, Kyoto, Japan) was employed to characterize structural information of the samples. UV–visible diffuse reflectance spectroscopy (UV-vis DRS, UV-2600) was measured to study the band-gap energies of the samples. The surface area and porosity analyzer (ASAP2460, Micromeritics, Norcross, GA, USA) were used to investigate the pore size distribution and specific surface area by Barrett–Joyner–Halenda (BJH) model and Brunauer-Emmett-Teller (BET) method, respectively.

### 2.3. Sensor Fabrication and Test

**Figure 2:**
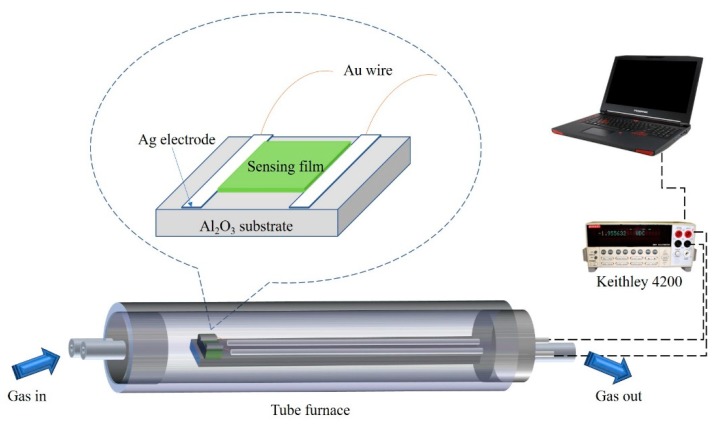
Illustration of gas sensor structure and test device.

A home-made system [30] was used to characterize the sensor. In brief, the sensing film of N-incorporated or pure SnO_2_ samples were formed by drop-coating on the substrate of Al_2_O_3_ with two Ag electrodes, whose two ends were connected with Au wires. The structure of the sensor and test device are shown in Figure 2. For the fabrication of Ag electrode, Ag paste was first printed on the Al_2_O_3_ substrate and Au wires and then dried by irradiation under an infrared lamp, which made Au wires, Ag powder, and the Al_2_O_3_ substrate stick together. All of them were heated at 550 °C for 30 min to sinter together. Since the work function of SnO_2_ studied in this work is ~3.1–3.6 eV, Ag seems to be the best electrode for conductive contact due to the high work function of Au and Pt. The obtained sensor was then put in to a quartz chamber for the test of the sensitivity. Before the test, the sensor was preheated and stabilized at 400 °C for 20 h to achieve good ohmic contact. The linear V-I curves of the N-incorporated sample on Ag electrodes coated Al_2_O_3_ substrate at 300 °C clearly imply good ohmic contact of the sensing film and the metal electrode (Figure S1). When the test began, the quartz chamber was fulfilled with target gas for ~0.65 min with 600 mL min^−1^ gas flow. The target gas was introduced into the quartz tube by mixing the certified gas “mixtures” (Beijing Hua Yuan Gas Chemical Industry Co., Ltd., Beijing, China) and dry air in a proper ration controlled by the mass flow controllers (CS-200C, Beijing Sevenstar Qualiflow Electronic Equipment Manufacturing Co., Ltd., Beijing, China). The bias on the sensor was set to 5 V. Keithley 4200 Sourcemeter was used to record the current. The response was defined as the ratio of sensor resistance in air and in the detected gas (R_air_/R_gas_ − 1), in which R_air_ and R_gas_ represent the electrical resistance of the sensor in air and test gas, respectively. The response/recovery time is defined as the time required for the resistance of the sensor to change to 90%/10% of the saturation value after exposure to the test gas/air. The coefficient of variation (CV) is used to represent the repeatability of the sensor, which is defined as

C_V_ = R_SD_/R_average_ × 100%, (1)

where R_SD_ and R_average_ are the standard deviation (SD) and average value of responses, respectively.

## 3. Results and Discussion

### 3.1. Morphology and Structural Characterization

**Figure 3:**
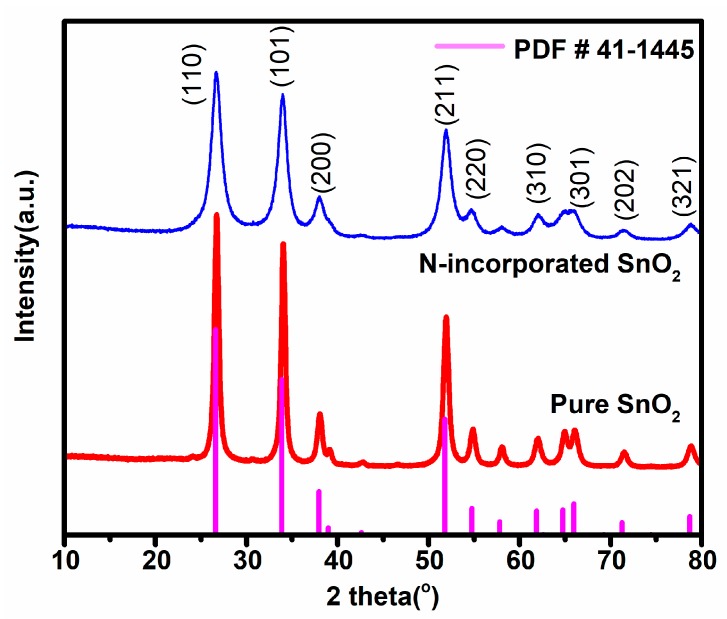
The XRD patterns of N-incorporated SnO_2_ and pure SnO_2_ samples.

The XRD patterns of N-incorporated SnO_2_ and pure SnO_2_ samples were shown in Figure 3. All diffraction peaks of N-incorporated SnO_2_ sample matched well with the standard diffraction data (PDF. No. 41-1445) for rutile structure SnO_2_. Comparatively, the diffraction peak positions of the pure SnO_2_ sample remained unchanged, while the diffraction peaks were apparently sharpened and the peak intensity became stronger. It indicates relatively higher crystallinity and larger crystallite size of the pure SnO_2_ sample than those of the N-incorporated SnO_2_ sample. In both samples, no other diffraction peaks of impurities phase were found. Based on the data of the (101) diffraction peaks, the average crystallite sizes of both samples were calculated by Scherrer’s formula [31]; these were 12 nm for the N-incorporated SnO_2_ sample and 30 nm for the pure SnO_2_ sample, respectively. 

**Figure 4:**
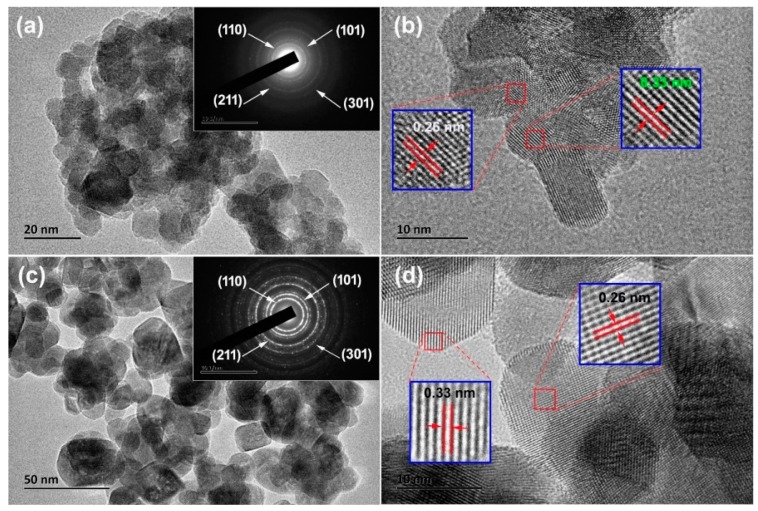
Transmission electron microscopy (TEM) and high-resolution TEM (HRTEM) images of the samples: TEM image (**a**) and HRTEM image (**b**) of N-incorporated SnO_2_ sample; TEM image (**c**) and HRTEM image (**d**) of pure SnO_2_ sample. The insets in (**a**) and (**c**) show the SEAD patterns of the samples.

To study the morphology and microstructures of N-incorporated SnO_2_ and pure SnO_2_ samples, TEM, high-resolution TEM (HRTEM), and selected area electron diffraction (SAED) patterns were measured, as shown in Figure 4. Figure 4a shows that the N-incorporated SnO_2_ sample was composed of nanoparticles of 10–20 nm in diameter, which were aggregated together to form porous structure. Comparatively, as shown in Figure 4b, the particle size of nanoparticles of the pure SnO_2_ sample was increased to 15–40 nm in diameter. The pore sizes of the pure SnO_2_ sample were also apparently larger than that of the N-incorporated SnO_2_ sample. The phenomenon of particle size reduction in SnO_2_ nanoparticles induced by nitrogen incorporation was also reported by Wang et al., which could be due to the suppression role of nitrogen atoms in the long-range order formation of host lattices [32]. The SAED pattern of the N-incorporated SnO_2_ sample (inset in Figure 4a) was featured by diffuse diffraction rings, as compared with distinct polycrystalline diffraction rings with some bright spots in the pure SnO_2_ sample (inset in Figure 4b). HRTEM images (Figure 4c,d) of both samples reveals distinct lattice lines with spacings of 0.33 and 0.26 nm, which are attributed to the (110) and (101) planes of rutile structure SnO_2_ [33]. Thus, both samples were well crystallized, which is consistent with the XRD results.

**Figure 5:**
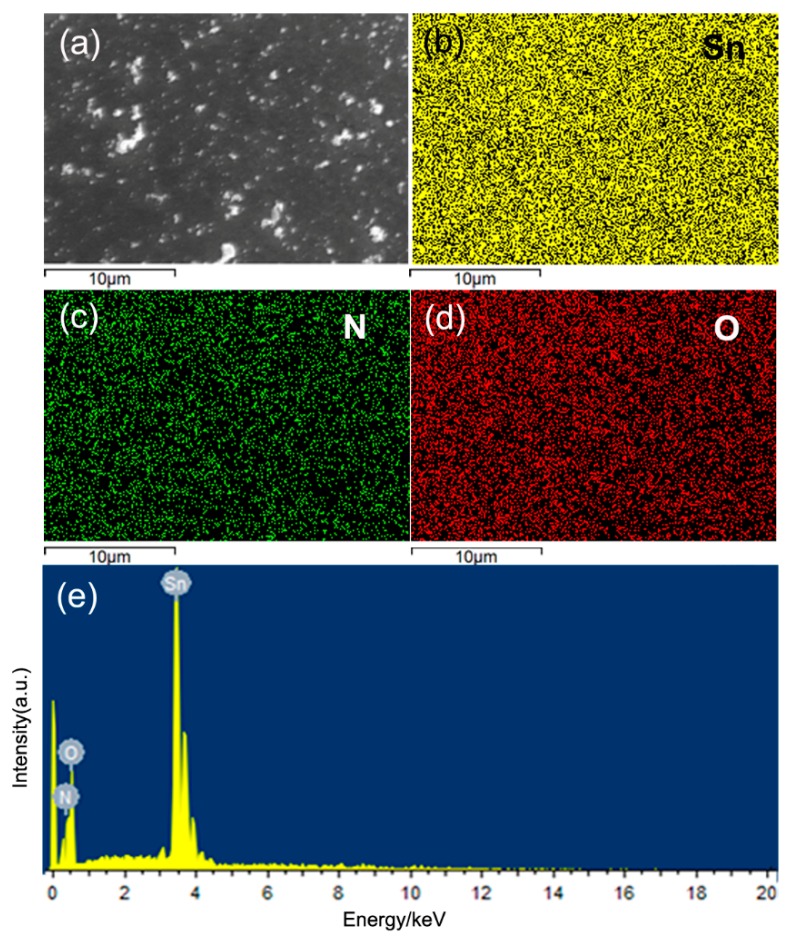
(**a**) Scanning electron microscopy (SEM) image and X-ray mapping of (**b**) Sn, (**c**) N, (**d**) O, and (**e**) energy dispersive spectrometry (EDS) spectrum of the N-incorporated SnO_2_ sample.

**Figure 6:**
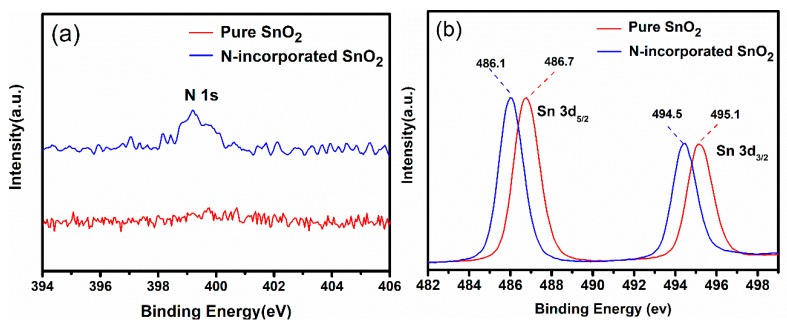
X-ray photoelectron spectroscopy XPS spectrum of (**a**) N 1s and (**b**) Sn 3d of N-incorporated SnO_2_ and pure SnO_2_ samples.

In order to verify the existence of the nitrogen incorporation in SnO_2_ nanoparticles, we carried out a detailed study on elements distribution and chemical compositions by EDS and XPS. As shown by EDS mapping results in Figure 5, only elements Sn, N, and O were found and they were homogeneously distributed in the N-incorporated SnO_2_ samples. Since no impurity phases were found in XRD detection, it indicates that nitrogen atoms could be incorporated into SnO_2_. The nitrogen incorporation is also confirmed by XPS analyses (Figure 6). A peak at 399.3 eV of N1s was only observed in N-incorporated SnO_2_ sample (Figure 6a), which confirmed the nitrogen incorporation into the surface of the sample [34]. Quantitative results show that the N content was 1.79 at% in N-incorporated SnO_2_ sample. Figure 6b shows the Sn 3d spectra of N-incorporated and pure SnO_2_ samples. For pure SnO_2_ sample, the peaks at 486.7 eV and 495.1 eV were identified to the standard Sn 3d5/2 and Sn 3d3/2 peaks of rutile SnO_2_, respectively. It indicates the existence of Sn^4+^ [35]. The difference of Sn 3d5/2 and Sn 3d3/2 peaks is 8.4 eV, which is in agreement with that in the literatures [36]. However, for N-incorporated SnO_2_ sample, the core binding energy of Sn 3d5/2 and Sn 3d3/2 peaks shifted toward lower binding energy by 0.6 eV. Such blueshift to lower binding energy has been reported in nitrogen-incorporated SnO_2_ films [37] and defective black SnO_2_ [38]. It could be ascribed to (i) the shift of 3d orbital energy level resulted from N^3−^ that has higher coulombic potential than O^2−^ [37]; and (ii) the formation of oxygen vacancies caused by charge compensation effect, which resulted in fewer O neighbours around Sn on average [38].

**Figure 7:**
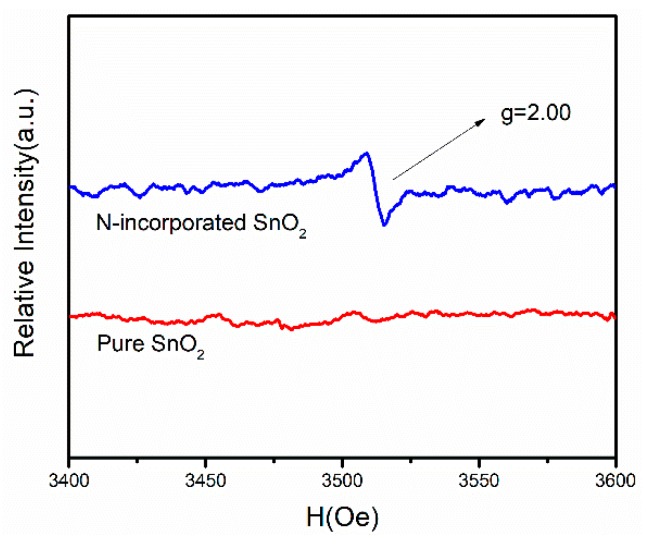
Electron paramagnetic resonance (EPR) signals of N-incorporated SnO_2_ and pure SnO_2_ samples.

The existence of oxygen vacancies in N-incorporated SnO_2_ sample was confirmed by the EPR signals. As shown in Figure 7, N-incorporated SnO_2_ sample exhibited a strong signal at g = 2.00, which is attributed to the electrons trapped on oxygen vacancies. In contrast, no apparent signal for pure SnO_2_ sample was observed. Thus, nitrogen incorporation is indicated to promote the formation of oxygen vacancies. Because the radius of nitrogen atom is close to that of the oxygen atom, the nitrogen atom can replace the oxygen atom. Since the nitrogen atom is trivalent and the oxygen atom is divalent, the doping of two nitrogen atoms will theoretically form an oxygen vacancy. Moreover, it is reported that nitrogen doping also reduces the formation energy of oxygen vacancies [39], which is also beneficial to the increase in oxygen vacancy concentration.

**Figure 8:**
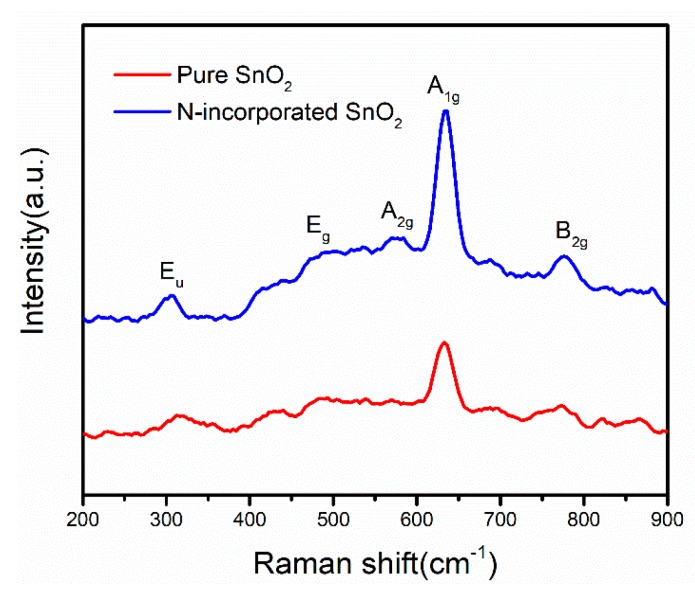
Raman spectra of the N-incorporated SnO_2_ and pure SnO_2_ samples.

In order to obtain more detailed information of oxygen vacancies, Raman spectra were further performed to study the structure difference between N-incorporated SnO_2_ and pure SnO_2_ samples, as shown in Figure 8. It is well known that the normal lattice vibration modes of rutile structure SnO_2_ at the Γ point of the Brillouin zone are given by

Γ = 1A_1g_ + 1A_2g_ + 2A_2U_ + 1B_1g_ + 1B_2g_ + 2B_1U_ + 1E_g_ + 4E_U_, (2)

Among these lattice vibration modes, three modes of A_1g_, B_2g_, and E_g_ are Raman active. Other modes including A_2g_, A_2u_, B_1g_, B_1u_, and E_u_ are Raman inactive because they do not appear in the single-crystal SnO_2_ according to symmetry analysis [40]. It was observed that both N-incorporated SnO_2_ and pure SnO_2_ samples clearly exhibited three characteristic vibration modes of A_1g_, B_2g_, and E_g_, which confirmed the rutile structure of SnO_2_. We noticed that the Raman inactive vibration modes of E_u_ and A_2g_ were also observed, which were located at around 300 and 578 cm^−1^, respectively. The appearance of these inactive vibration modes has been ascribed to the existence of oxygen vacancies in rutile SnO_2_ [41]. Notwithstanding similar spectral features in both samples, the intensities of A_1g_, A_2g_, and E_u_ peaks appeared to be much stronger in N-incorporated SnO_2_ sample in comparison with the pure SnO_2_ sample. It is well documented [42] that oxygen vacancies in SnO_2_ could be classified as three types of subbridging, bridging, and in-plane oxygen vacancies, which are responsible for E_u_, A_1g_, and A_2g_ modes, respectively. The peak intensities of these modes are proportional to the density of oxygen vacancies [43]. Thus, the incorporation of N atoms into SnO_2_ apparently increased the concentration of these oxygen vacancies, which is well consistent with EPR result. Additionally, it is noted that the Eu peak exhibits a decrease in wavenumber (redshift) in N-incorporated SnO_2_ sample. It has been widely accepted that oxygen has a Pauling electronegativity of 3.5, which is larger than that of 3 for nitrogen. Thus, the ability of an oxygen nucleus to attract electrons from the Sn atom is stronger than that of N atom. Therefore, the substitution of N^3−^ for O^2−^ would result in the increase of electron density around Sn atom in the O-Sn-O structure, as well as the formation of oxygen vacancies ascribed to the charge compensation effect. In consequence, the spatial charge between O and Sn could be redistributed to weaken the strength of the Sn–O bond, leading to the redshift of the E_u_ peak. Thus, above results provide an insight into the impact of nitrogen incorporation in the Raman behaviour of oxygen vacancies in SnO_2_ nanomaterials.

**Figure 9:**
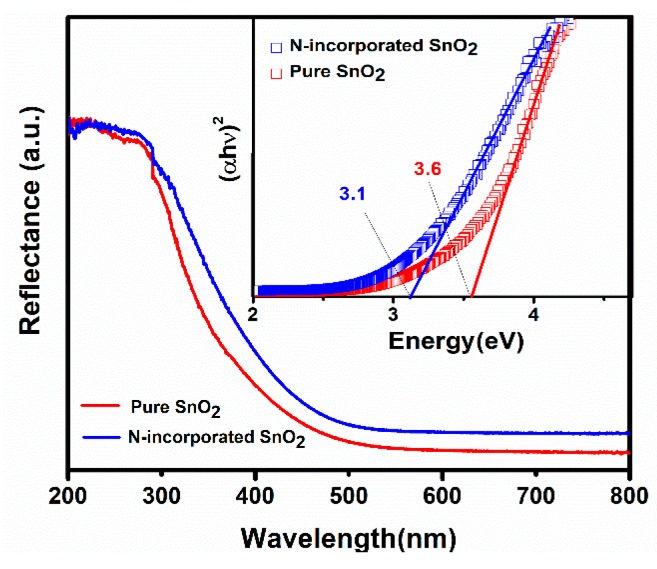
Optical diffuse reflectance spectra of the N-incorporated SnO_2_ and pure SnO_2_ samples. The inset shows the energy dependence of (αhν)^2^ for the samples.

To investigate the influence of nitrogen incorporation and oxygen vacancies on the band-gap width of rutile SnO_2_, UV–visible diffuse reflectance spectroscopy of N-incorporated SnO_2_ and pure SnO_2_ samples were performed. As shown in Figure 9, the absorption edge of N-incorporated SnO_2_ sample shifts to a longer wavelength as compared to that of pure SnO_2_ sample. Such a red-shift phenomenon is consistent with other experimental findings [37]. The band-gap energy can be calculated by the following equation:

α*h*ν = *A* (*h*ν − *E_g_*)*^n^*, (3)

where α is the absorption coefficient, *E_g_* is the band-gap energy, *h*ν is the photon energy, *n* equals 0.5 for direct allowed transition or 2 for indirect allowed transition, respectively. Since SnO_2_ is a direct type semiconductor, *n* equals 0.5 in this case. Thus, the value of *E_g_* can be obtained through extrapolating the linear portion towards zero absorption by fitting the plot of (α*h*ν)^2^ as a function of *h*ν, as shown in the inset in Figure 9. It is seen that the value of *E_g_* for pure SnO_2_ is 3.6 eV, similar to the reported value for bulk SnO_2_ [44], while the value of *E_g_* for N-incorporated SnO_2_ was decreased to 3.1 eV. Since it was well documented that the reduction of particle size could increase the band gap of SnO_2_ [45], we believe that the nitrogen incorporation is the main reason for the decrease of the band-gap of N-incorporated SnO_2_ sample. In this regard, Sun et al. [28] have performed the density functional theory (DFT) calculations of nitrogen doping behaviours on the single-crystalline rutile SnO_2_. Their calculation has drawn a conclusion that the N atom is energetically favourable to be incorporated to the O site. As N atom is incorporated, the N 2p states are delocalized and contributory to the formation of some gap states hybridized by O 2p states and Sn 3d states, which results in a band to gap states transition and causes the reduction of the band-gap.

**Figure 10:**
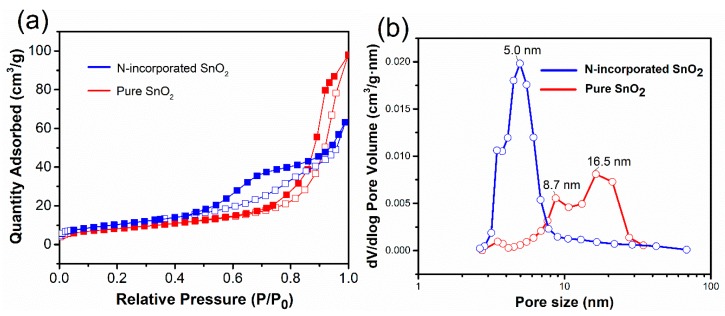
(**a**) N_2_ adsorption-desorption isotherm curves and (**b**) pore size distribution curves of the N-incorporated SnO_2_ and pure SnO_2_ samples.

The impacts of N incorporating on the surface areas and pore distribution of the samples were investigated by the surface area and porosity analyzer. The surface area of N-incorporated SnO_2_ and pure SnO_2_ samples were calculated by BET method, which are 38.2 and 29.7 m^2^/g, respectively. The absorption–desorption isothermals of N-incorporated SnO_2_ and pure SnO_2_ samples are shown in Figure 10a. As can be seen, N-incorporated sample shows a typical IV type isotherm with a H2 hysteresis loop as classified by International Union of Pure and Applied Chemistry (IUPAC) [46], which is a characteristic of mesoporous system [47]. The sharp infection of the hysteresis loop is located at a range of P/P_0_ =0.6–0.8. Comparatively, pure SnO_2_ samples show a similar type isotherm, while the sharp infection of hysteresis loop shifted toward higher P/P_0_ value. It indicates that the pore diameter of the mesoporous system increased. The pore size distribution curves for the mesoporous structures were determined from the desorption branches using the BJH model [48], as shown in Figure 10b. The N-incorporated SnO_2_ sample exhibited a narrow pore size distribution centered at about 5 nm, while pure SnO_2_ sample showed a broad pore size distribution centered at 8.7 and 16.5 nm. Therefore, the N-incorporated SnO_2_ sample exhibits larger surface area and smaller mesopores than pure SnO_2_ sample does, which may be beneficial to accelerating the adsorption and diffusion of target gas.

### 3.2. Gas sensing Properties

**Figure 11:**
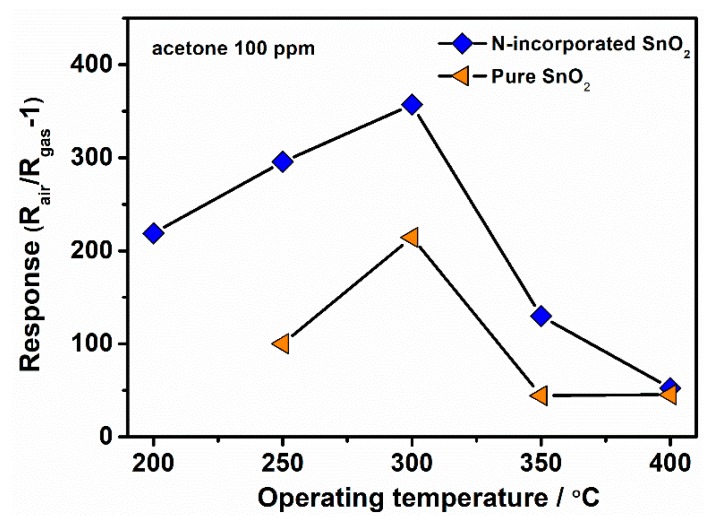
Temperature dependent responses comparison of N-incorporated SnO_2_ and pure SnO_2_ gas sensors.

According to the increased oxygen vacancies concentration and mesoporous structure of the N-incorporated SnO_2_ sample, we anticipate that it may probably possess good gas sensing properties. Sensor measurements were explored by using acetone as the target gas. We firstly studied the influence of working temperature on the sensor response of the N-incorporated SnO_2_ sample to 100 ppm acetone gas, as well as that of pure SnO_2_ sample for comparison, as shown in Figure 11. It can be seen that the sensor response of N-incorporated SnO_2_ sample initially increased with the increasing of working temperature, and reached to a maximum response (R_air_/R_gas_ − 1 = 357) at 300 °C, and finally decreased with further increase of working temperature. Comparatively, the pure SnO_2_ sample showed similar temperature dependent responses to 100 ppm acetone, except that its maximum response is only 215 at 300 °C. As shown in Figure S3, the poor response speed (>3 min) and the failure to recover to 10% of the resistance change clearly indicate that temperature higher than 200 °C is necessary for the N-SnO_2_ sample. It has been reported that higher temperature can provide thermal energy for the reaction of the target gases and oxygen ions on the surface of SnO_2_, which is responsible for the increase of gas response with the increasing working temperature. At the same time, higher temperature can promote the desorption of oxygen ions from the surface of SnO_2_, which reduce the gas response of SnO_2_. In this work, the role of thermal energy is dominant below 300 °C, while the desorption of oxygen ions plays more significant role above 300 °C. In addition, it seems that nitrogen incorporation has no apparent impact on the optimal working temperature of SnO_2_. Thus, the optimal working temperature of 300 °C is applied in the subsequent gas sensing measurements.

**Figure 12:**
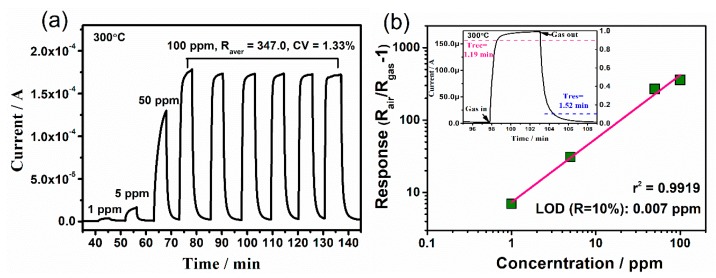
(**a**) Typical response-recovery current curves toward acetone gas with different concentration, (**b**) log-log plots of concentration-responses of N-incorporated SnO_2_ gas sensor (the inset is the response and recovery time for the curve toward 100 ppm of acetone).

Figure 12a shows the dynamic responses of N-incorporated SnO_2_ sample to acetone gas with different concentrations in dry air (1~50 ppm) and to 100 ppm acetone with six successive assays. The current curve presents good response-recovery to a broad range of the acetone concentrations (1 to 100 ppm) and typical behaviours of n-type semiconductor chemiresistor gas sensor. Notably, low C_V_ (1.33%) of six circles can be estimated, implying excellent repeatability of the device. According to the response equation of grain-based gas sensors and R = R_air_/R_gas_ − 1, we can obtain the following equation (for resistance decrease) [49]:

logR = log(R_air_/R_gas_ − 1) = logA_g_ + βlogp_g_, (4)

where p_g_ is the gas partial pressure, A_g_ is a prefactor, and the exponent β is the response order.

The limit of detection (LOD) as low as 0.007 ppm (7 ppb) of acetone was estimated according to Equation (4) by setting R = 0.1 for N-SnO_2_ (Figure 12b). Satisfying values of response time and recovery time can be obtained, which were calculated to be 1.19 min and 1.52 min, respectively. It is found that recovery time obviously decreased with the increasing temperature, as shown in Figure S2. It is because that the additional thermal energy facilitates the adsorption/desorption of target gas molecules, thus good recovery at higher temperature can be accordingly observed.

**Figure 13:**
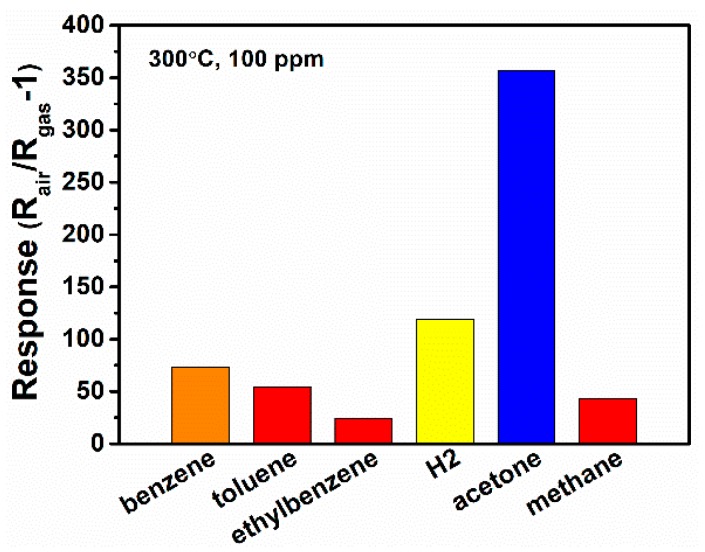
Cross-sensitivity toward 100 ppm of various reducing gas at 300°C of N-incorporated SnO_2_ gas sensor.

Furthermore, the selectivity of the sensor is evaluated by the cross-sensitivity measurement for a series of reducing gas (100 ppm for each) at 300 °C, as shown in Figure 13. For the indoor air monitoring and breath analysis, benzene and alkanes are two typical interferences for acetone sensing. Thus, we choose other reducing gas including benzene, toluene, ethylbenzene, hydrogen, and methane as the interfering gases. It can be found that the N-incorporated SnO_2_ sample shows much higher sensibility to acetone than other reducing gas including benzene, toluene, ethylbenzene, hydrogen, and methane, which indicate good selectivity of the N-incorporated SnO_2_ sample toward acetone gas. The stability of the N-incorporated SnO_2_ sample towards 100 ppm acetone at 300 °C was evaluated over a week period, as shown in Figure S4 in supporting information. The results show that the gas sensor maintains 97% of its original response to acetone. Thus, the N-incorporated SnO_2_ sample is expected to have good long-term stability. The repeatability within a batch of three samples is good with the relative standard deviation (RSD) of 2%, as shown in Table S1.

For comparison, the sensing abilities of as-prepared N-incorporated SnO_2_ and other reported SnO_2_ nanostructures are listed in Table 1. It can be seen that N-incorporated SnO_2_ in this work exhibits outstanding gas sensing performance with the highest sensor response (R_air_/R_gas_ − 1) value of 357 and the lowest LOD value as 0.007 ppm (7 ppb) at 300 °C, demonstrating more superiority than those reported in the literature. Considering the low power consumption towards practical application, further experiments on developing low-temperature (<200 °C) N-incorporated SnO_2_ gas sensors still need to be done in our future work. There are several good strategies such as the introduction of heterostructures [50], morphologies controlling [51], surface synergy [52,53], and molecule sieving layer coatings [49], etc.

### 3.3. Gas-Sensing Mechanism

It has been known that the gas-sensing mechanism of SnO_2_-based gas sensor is primary ascribed to the resistance change of SnO_2_ in different target gas atmospheres, which is caused by the adsorption and desorption of target gas molecules on the surface of SnO_2_. In air atmosphere, oxygen molecules are adsorbed on the surface of SnO_2_ and capture the electrons from the conduction band of SnO_2_, forming the surface adsorbed oxygen species such as O_2_^−^ and O^−^. This progress could be described as follows:

(5) $$O_{2}\left(\mathrm{gas}\right)↔O_{2}\left(\mathrm{ads}\right),$$

(6) $$O_{2}\left(\mathrm{ads}\right)+e^{-}↔O_{2}^{-}\left(\mathrm{ads}\right),$$

(7) $$O_{2}^{-}\left(\mathrm{ads}\right)+e^{-}↔2O^{-}\left(\mathrm{ads}\right),$$

Under this condition, the concentration of free electrons in the conduction band of SnO_2_ decreased, forming the electron depletion layer and potential barrier on the surface and at grain boundaries, respectively. As a result, the sensor resistance will increase vastly. The potential barrier could be described by Equation (8) [73]:

(8) $$\varphi_{B}=AN_{d}W_{D}^{2},$$

where $\varphi_{B}$ is the potential barrier, $N_{d}$ is the donor density, *W_D_* is the width of depletion layer, and *A* is the constant. When in a condition of reducing gas of acetone, the surface oxygen species of SnO_2_ would react with acetone and free the trapped electrons back into the conduction band of SnO_2_, which would reduce the width of the depletion layer and thus lower the height of potential barrier. Consequently, the sensor resistance will decrease. The possible reaction is shown in Equation (9) [74].

CH_3_COCH_3_ (ads) + 8O^−^ (ads) → 3CO_2_ (gas) + 3H_2_O (liq) + 8e^−^ (9)

**Figure 14:**
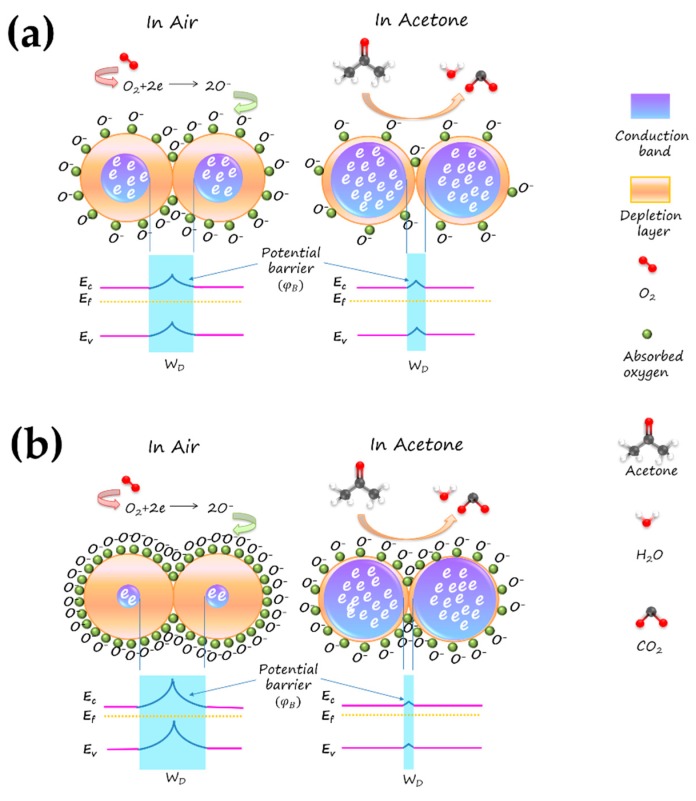
Schematic of acetone sensing mechanism of (**a**) pure SnO_2_ and (**b**) N-incorporated SnO_2_ samples.

Having aforementioned discussion in mind, we believe that the significantly enhanced gas-sensing property of the N-incorporated sample should be mainly attributed to the following three main reasons. Firstly, the incorporation of nitrogen into SnO_2_ resulted in rich surface oxygen vacancies. These surface oxygen vacancies tend to adsorb oxygen molecules, because of the lower adsorption energy of oxygen molecules on the oxygen vacancy sites than that on the perfect sites [60,75]. Therefore, these surface oxygen vacancies can act as electron donors, making a great quantity of electrons in the conductive band captured in air and released in acetone atmosphere. It would greatly increase the difference of the width of depletion layer (*W_D_*) and the height of potential barrier ($\varphi_{B}$) in different atmospheres, resulting in a higher response. Secondly, as N atom is incorporated, the N 2p states are delocalized and contributory to the formation of some gap states hybridized by O 2p states and Sn 3d states, which results in a band to gap states transition and causes the reduction of the band gap. It makes electrons more easily excited, which improves electron transport. Thirdly, the incorporation of nitrogen into SnO_2_ resulted in the reduction of particle size, along with the large surface area and unique mesoporous structure. These would provide more active sites on the surface of SnO_2_, and facilitate acetone diffusion and mass transport within sensing material. Therefore, the N-incorporated SnO_2_ shows a superior gas sensing property. The gas-sensing mechanism of as-prepared N-incorporated SnO_2_ sensor is shown in Figure 14.

## 4. Conclusions

In summary, mesoporous N-incorporated SnO_2_ and pure SnO_2_ nanostructures have been prepared by simple solvothermal and calcination procedure. The XRD and TEM results showed that the N incorporating led to the decreased crystalline size of SnO_2_, without changing the rutile crystal structure. EDS and XPS analysis confirmed the presence of nitrogen in the N-incorporated SnO_2_ nanostructure. It resulted in obviously increased surface oxygen vacancies, which has been revealed by EPR and Raman. It has been also observed that N-incorporated SnO_2_ nanostructure exhibited reduced band-gap width, larger surface area, and smaller mesopore size, in comparison with pure SnO_2_. The gas sensor based on N-incorporated SnO_2_ nanostructure exhibited excellent acetone gas-sensing property with high sensor response (R_air_/R_gas_ − 1 = 357) and low limit of detection (7 ppb) at the optimal operating temperature of 300 °C. Moreover, the N-incorporated SnO_2_ gas sensor shows a good selectivity to acetone in the interfering gases of benzene, toluene, ethylbenzene, hydrogen, and methane. The enhancing role of N incorporation could be attributed to increasing the surface oxygen vacancies, reducing band-gap width, and lowering the crystal size of nanoparticles. Hence, N-incorporated SnO_2_ nanostructure could be a promising candidate material for highly sensitive gas sensor toward acetone gas. Our future studies will focus on the low-temperature N-incorporated SnO_2_ gas sensor to meet the demand of low power consumption towards practical application.

## Figures and Tables

**Table 1:** Gas responses to acetone of as-prepared N-incorporated SnO_2_ and other reported SnO_2_ nanostructures.

Sensing Materials	Acetone Concentration (ppm)	Working Temperature (°C)	Sensor Response (R_air_/R_gas_ − 1)	LOD	Ref.
SnO_2_ nanoparticles	100	240	17	0.2 ppm ^E^	[54]
SnO_2_ hollow microspheres	160	200	30	5 ppm ^E^	[55]
SnO_2_ nanoployhedrons	100	370	29	1 ppm ^E^	[56]
α-Fe_2_O_3_/SnO_2_ composites	100	250	15.8	10 ppm ^E^	[57]
γ-Fe_2_O_3_@SnO_2_ nanoparticles	100	370	5	10 ppm ^E^	[58]
Fe-incorporated SnO_2_	100	200	29	0.1 ppm ^E^	[59]
Eu-incorporated SnO_2_ nanofibers	100	280	31.2	0.3 ppm ^E^	[60]
Ca^2+^/Au co-incorporated SnO_2_	100	200	61	NM	[61]
cone-shaped SnO_2_	100	325	174	NM	[62]
Ce-incorporated SnO_2_	100	270	99	NM	[63]
Pd loaded Sm incorporated SnO_2_	100	200	15.7	NM	[64]
SnO_2_ nanospike arrays	100	320	39	0.5 ppm ^E^	[65]
SnO_2_ Hollow nanospheres	100	400	7.5	NM	[66]
Rh-incorporated SnO_2_ nanofibers	100	200	132	NM	[67]
C_3_N_4_-SnO_2_	100	380	28 ^a^	0.067 ppm ^C^	[68]
In loaded WO_3_/SnO_2_	100	200	129	NM	[69]
SnO_2_/Au-incorporated In_2_O_3_	100	280	11.4	NM	[70]
Ag/SnO_2_ hollow nano fibers	100	200	74	NM	[71]
Nanofibrous Pd-loaded SnO_2_	100	275	97.8	NM	[72]
N-incorporated SnO_2_	100	300	357	0.007 ppm ^C^	TW

^a^ Response calculated as V_g_/V_a_; ^C^ Calculated value; ^E^ measured value; NM: not mentioned; TW: this work.

## Supplementary Materials

The following are available online at https://www.mdpi.com/2079-4991/9/3/445/s1, Figure S1: The V-I curve of the N-incorporated SnO_2_ sample on Ag electrodes coated Al_2_O_3_ substrate at 300 °C; Figure S2: Typical response-recovery current curves of the N-incorporated SnO_2_ gas sensor toward acetone gas with different concentration at different temperatures: (a) 250 °C; (b) 350 °C; (c) 400 °C; Figure S3: The response and recovery curve for the N-incorporated SnO_2_ sample toward 1 ppm of acetone at 200 °C; Figure S4: Stability response curve of the N-incorporated SnO_2_ gas sensor toward 100 ppm of acetone at 300 °C. Table S1: The repeatability within a batch of three samples.
