# Supplementary material for: Incorporating N Atoms into SnO2 Nanostructure as an Approach to Enhance Gas Sensing Property for Acetone
Source: Nanomaterials (Basel). 2019 Mar 15;9(3):445. doi: 10.3390/nano9030445 (PMC6474091; doi:10.3390/nano9030445)
Supplement: Supplementary file 1 [file nanomaterials-09-00445-s001.pdf]

Supporting Information:

Incorporating N atoms into SnO_2 nanostructure as an approach to enhance gas sensing property for acetone

Xiangfeng Guan [1,*], Yongjing Wang [2], Peihui Luo [1], Yunlong Yu [1], Dagui Chen [1], and Xiaoyan Li [1]

[1] Organic Optoelectronics Engineering Research Center of Fujian's Universities, Fujian Jiangxia University, Fuzhou 350108, P.R. China; xfguan@fjjxu.edu.cn(X.G.); phluo@fjjxu.edu.cn (P. L.); ylyu@fjjxu.edu.cn (Y.Y); dgchen@fjjxu.edu.cn (D.C.); xyli@fjjxu.edu.cn (X. L.).

[2] College of Environment and Resources, Fuzhou University, Fuzhou 350108, P.R. China; yjwang03@fzu.edu.cn

* Correspondence: xfguan@fjjxu.edu.cn (X.G.); Tel.: +86-591-2353-1652

Figure S1:

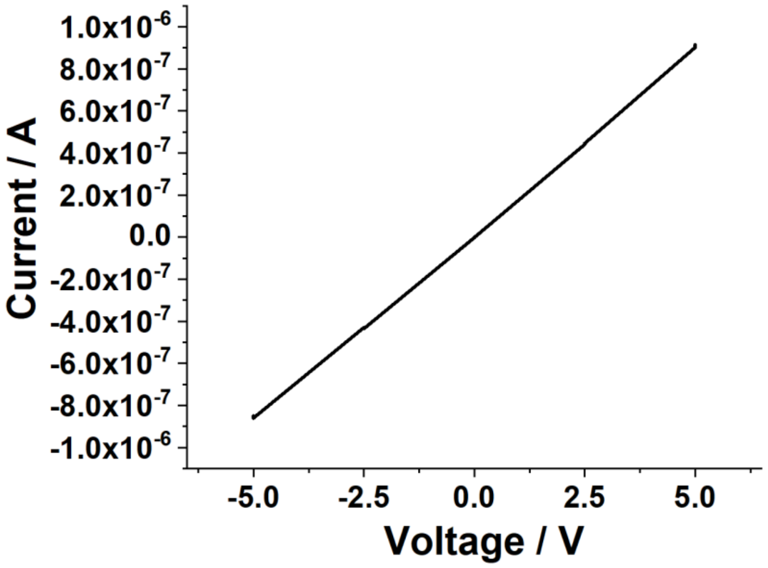

Figure S1. The V-I curve of the N-incorporated SnO_2 sample on Ag electrodes coated Al_2O_3 substrate at 300 °C

Figure S2:

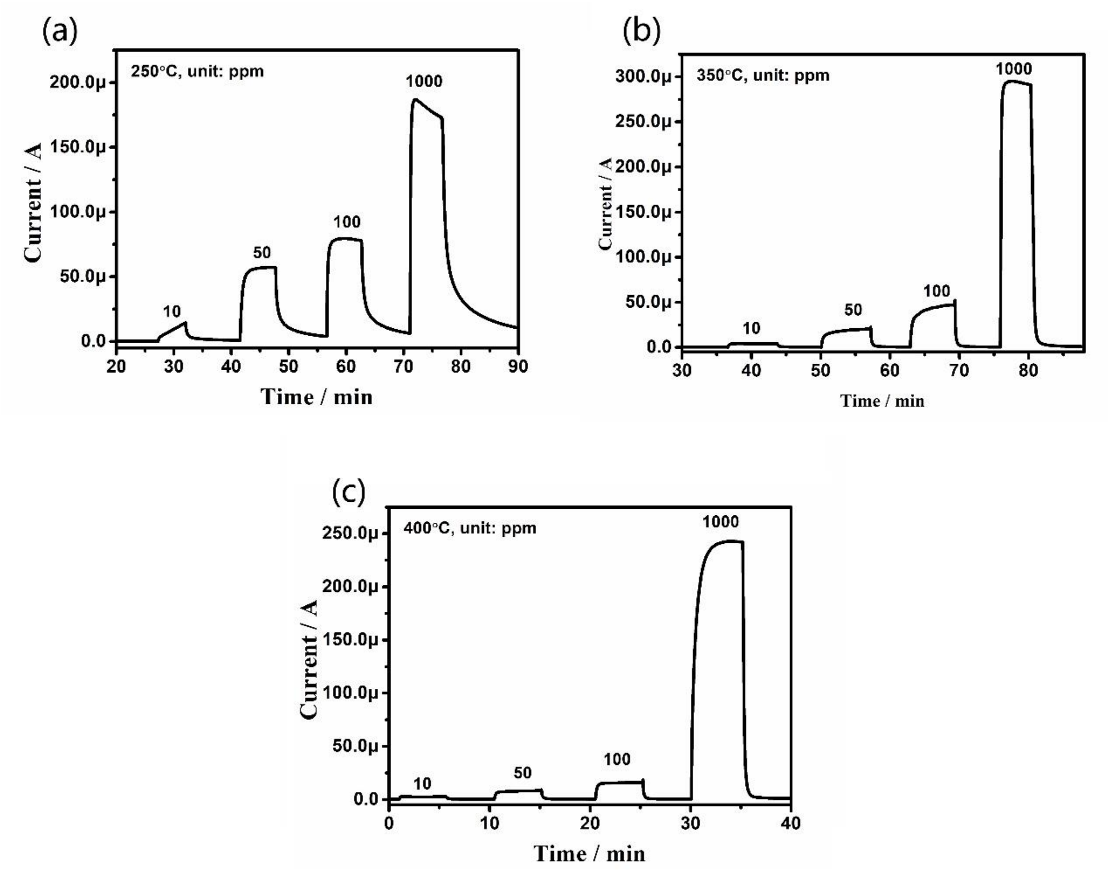

Figure S2. Typical response-recovery current curves of the N-incorporated SnO_2 gas sensor toward acetone gas with different concentration at different temperatures: (a) 250 ºC; (b) 350 ºC;(c) 400 ºC.

Figure S3:

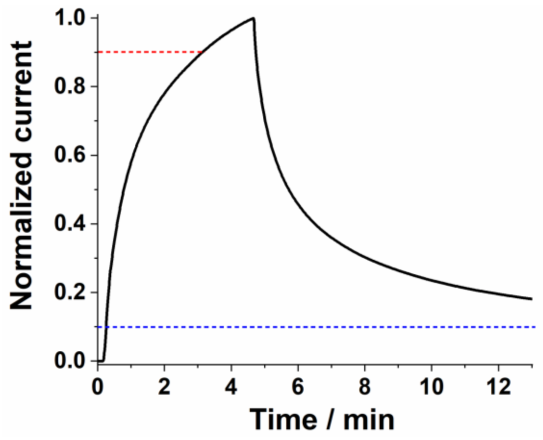

Figure S3. The response and recovery curve for the N-incorporated SnO_2 sample toward 1 ppm of acetone at 200 ºC

Figure S4:

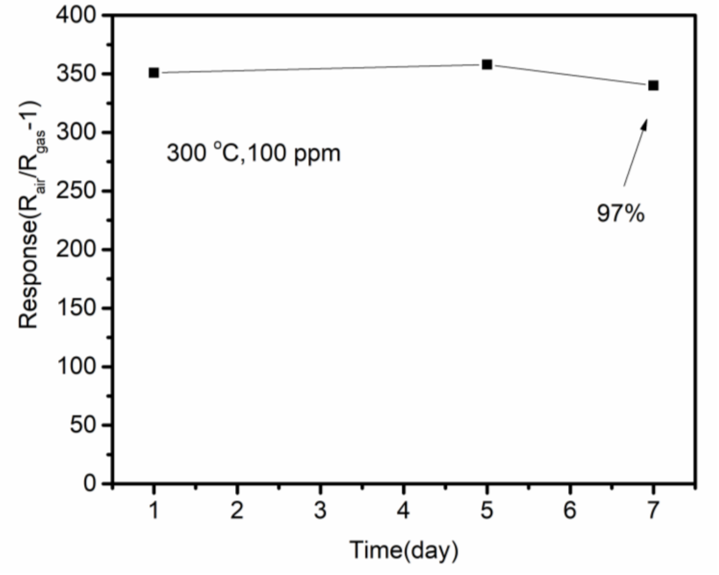

Figure S4. Stability response curve of the N-incorporated SnO_2 gas sensor toward 100 ppm of acetone at 300 °C.

Table S1: The repeatability within a batch of three samples

Sample number	Acetone concentration (ppm)	Working temperature (°C)	Sensor Response (R_{air}/R_{gas}-1)	Relative standard deviation (RSD)
1#	100	300	357	2%
2#	100	300	351	
3#	100	300	343	
